# Persuasive chatbot-based interventions for depression: a list of recommendations for improving reporting standards

**DOI:** 10.3389/fpsyt.2025.1429304

**Published:** 2025-06-19

**Authors:** Kerstin Denecke, Octavio Rivera Romero, Rolf Wynn, Elia Gabarron

**Affiliations:** ^1^ AI for Health, Institute Patient-centered Digital Health, Department Engineering and Computer Sciences, Bern University of Applied Sciences, Biel, Switzerland; ^2^ Instituto de Ingeniería Informática (I3US), Universidad de Sevilla, Sevilla, Spain; ^3^ Electronic Technology Department, Universidad de Sevilla, Sevilla, Spain; ^4^ Department of Clinical Medicine, Faculty of Health Sciences, UiT The Arctic University of Norway, Tromsø, Norway; ^5^ Department of Education, ICT and Learning, Østfold University College, Halden, Norway; ^6^ Norwegian Centre for E-health Research, University Hospital of North Norway, Tromsø, Norway

**Keywords:** chatbot, depression, natural language processing, guidelines, reporting

## Abstract

**Background:**

Depression is the leading cause of disability worldwide. Digital interventions based on chatbots could be an alternative or complementary approach to the treatment of depression. However, the absence of technical information in papers on depression-related chatbots often obstructs study reproducibility and hampers evaluating intervention efficacy.

**Objective:**

This study aims to identify specific characteristics of chatbots for depression and formulate recommendations for improving reporting standards.

**Methods:**

In an initial step, a list of items that must be reported was defined based on a previous review on digital interventions for depression, the Behavior Change Wheel framework, and a taxonomy for defining archetypes of chatbots. To capture the existing knowledge on the development of chatbots for depression, a literature review was conducted in a second step. From the identified studies, we tried to extract information related to the items from our initial list and described in this way the chatbots and their evaluation. As a third step, the findings of the literature review were analyzed, leading to an agreement on a list of recommendations for reporting chatbot-based interventions for depression.

**Results:**

The items of the recommendation list for reporting fall into four dimensions: General information; Chatbot-based depression intervention functions; Technical data; and Study. Through a literature review, a total of 23 studies on chatbots for depression were identified. We found that a lot of information as requested by our initial reporting list was missing, specifically regarding the involvement of natural language processing, data privacy handling, data exchange with third-party providers, and hosting. Additionally, technical evaluation details were often unreported in many papers.

**Conclusion:**

Studies on chatbots for depression can improve reporting by specifically adding more technical details and chatbot evaluation. Such reporting of technical details is important even in papers on clinical trials that utilize chatbots in order to allow reproducibility and advance this field. Future work could obtain expert consensus on the recommended reporting items for chatbot-based interventions for depression.

## 1 Introduction

Depression is a common mental disorder that affects approximately 280 million people in the world (1). It is also a leading cause of disability worldwide, and its impact has significantly intensified following the COVID-19 pandemic (2). Depression causes severe symptoms such as depressed mood or loss of pleasure or interest in activities for longer periods of time. It can impact all aspects of life of an individual including social relationships, school or work, and it can in some cases lead to suicide (3). Psychological interventions are effective to treat persons suffering from depression (4), especially behavioral activation, cognitive behavioral therapy, interpersonal psychotherapy or problem-solving therapy (5, 6). Typically delivered as talk therapy, these interventions help learning new ways of thinking, coping or relating to others.

Psychological interventions in the context of depression may be accessed through self-help manuals, websites and apps, and may include blended psychotherapy, which combines internet and mobile-based interventions in both outpatient and inpatient psychotherapeutic settings (7). This methodology enhances therapy by incorporating online treatment modules as an adjunct therapeutic tool, allowing patients to engage in interventions independent of time and place, thereby increasing the effectiveness and accessibility of face-to-face sessions. Serrano-Ripoll et al. studied the efficacy of app-based psychological interventions for reducing depressive symptoms in people with depression (8). They confirmed that apps can result in moderate reductions in the symptoms of depression, but also state that more studies are needed to determine which intervention features are associated with greater improvements.

In recent years, these digital interventions can be realized as chatbots, i.e. dialog-based systems with which a user can interact using natural language. Ahmed et al. reviewed 11 chatbot apps for anxiety and depression available in app stores (9). They found that such “apps provide a unique opportunity for cost effective alternative approaches to meet shortfall in health professionals”. Bendig et al. provided an overview on chatbots to foster mental health (10) and concluded that “the technology of chatbots is still experimental in nature”. Additionally, Ahmed et al. studied chatbot features for anxiety and depression (11) and found that most chatbots ​​follow a traditional way of therapeutic counseling and include cognitive behavior therapy. They collected technical features of these chatbots including input/output modality, initial dialogue technique or platform. However, they did not study important relevant technical features such as data processing aspects or how natural language processing including sentiment or emotion analysis is realized.

Denecke et al. introduced a technically oriented taxonomy for chatbots in healthcare (12) that was already suggested as a reporting guideline for studies on chatbots in healthcare. Its application for cluster analysis already showed that scientific papers on chatbots in healthcare are not providing all essential information to ensure transparency on technical implementation, including data processing and data security. The lack of technical information in papers on chatbots related to depression often hinders the reproducibility of studies and limits the evaluation of the efficacy of the digital interventions. This threat to the quality of the research makes it difficult to generate scientific evidence. The specific characteristics of chatbot-based intervention for depression might require the addition of new technical features to the general taxonomy proposed by Denecke and May (12). Additionally, we assume that use-case specific aspects related to the treatment of depressions must be reported to ensure reliability of scientific evidence. With this work, we want to identify the specific characteristics of chatbots for depression treatment and formulate a list of recommendations for reporting scientific studies that goes beyond reporting details on the study but focuses on the technical-related features. This resulting list of recommendations for reporting includes the relevant technical aspects of the chatbots, extending the taxonomy provided by Denecke and May (12), and the use-case specific aspects related to the treatment of depressions.

## 2 Methods

### 2.1 Method for establishing recommendations for reporting items

**Figure 1:**
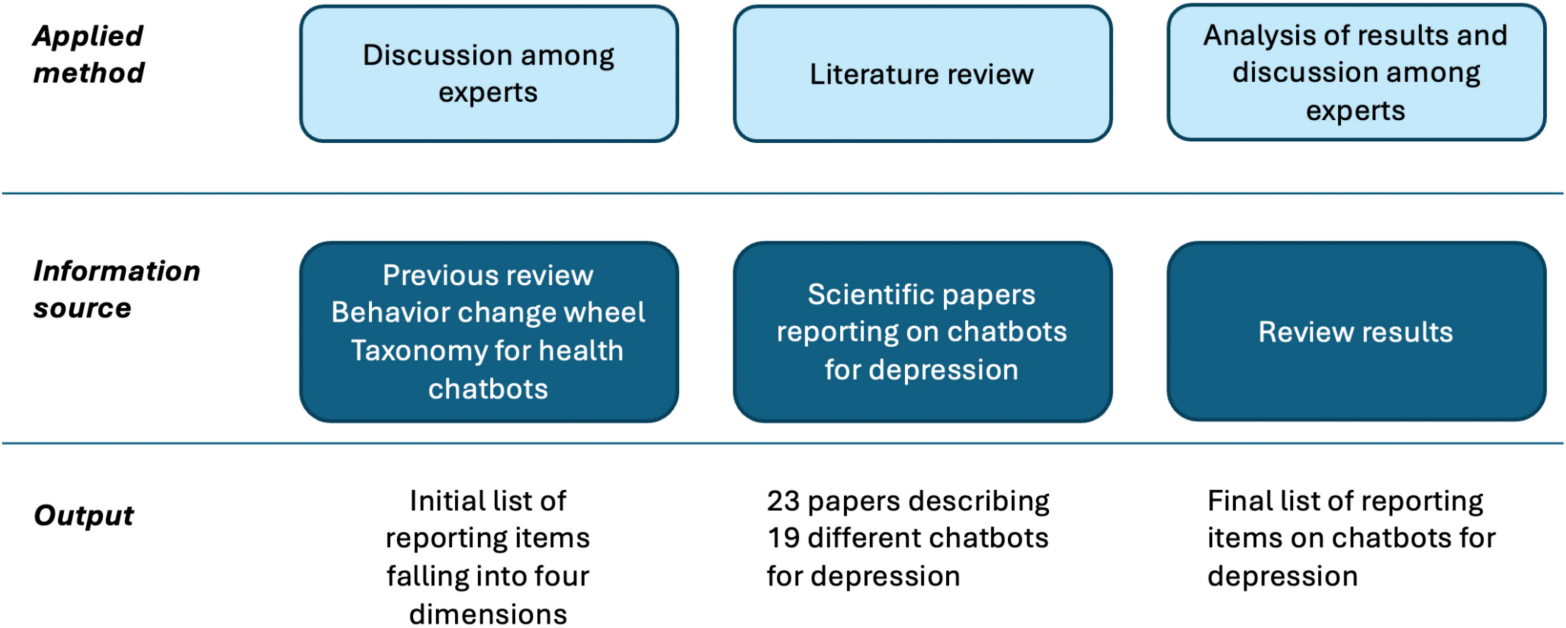
Overview on the methodology for developing recommendations for reporting on chatbots for depression.

We propose a 3-steps process to define a list of recommendations for reporting chatbot-based interventions for depression (see **Figure 1**). The objective of the first step is to define an initial list of items that must be reported in these studies. This initial list was defined based on a previous review (13), the Behavior Change Wheel framework (14), and the taxonomy proposed by Denecke and May (12). Authors participated in a joint meeting to discuss and agree on the items to be included in the initial list of recommendations based on their experience on interventions for depression and digital health, particularly in chatbot development and their evaluation. The items were structured along four main dimensions: General information; Study; Chatbot-based depression intervention functions; and Technical data. More information on this list is provided in section 3.1.

To study the applicability of our guideline and to capture the existing knowledge on the development of chatbots for depression, a literature review was conducted in the second step. This review followed the recommendations stated in PRISMA 2020 statement (15). The review methodology is described in section 2.2. Finally, in the third step, authors analyzed the findings of the literature review and they agreed on a list of recommendations on reporting chatbot-based interventions for depression.

### 2.2 Literature review

The search was carried out on January 8th 2024, and covered five databases: PubMed, ACM, IEEExplore, PsycInfo, and CINAHL. We searched for publications that in the title or abstract included keywords related to depression and chatbots. No year or language limitations were used for this search. A single reviewer did the search engine (ORR). The full search strategy is presented in the **Supplementary Material 1**. Identified publications were included in our review if they met the following inclusion and exclusion criteria.

Inclusion criteria:

- Describes primary research,
- Describes an already developed chatbot,
- The chatbot is used for an intervention for any of the following <2 conditions: Depression, Depressive disorder; Affective disorder; Dysthymia; or Dysthymic disorder.

Exclusion criteria:

- Reviews, study protocols, comments, patents and white papers.
- The chatbot is not described or it has not been developed.
- The chatbot is not used for an intervention for any of the following conditions: Depression, Depressive disorder; Dysthymia; Affective disorder; Dysthymic disorder.

All identified references were uploaded to EndNote 20 and Rayyan. After removing duplicates, a reviewer (EG) conducted the initial screening by reading titles and abstracts. A second reviewer (KD) verified 10% of the included and excluded articles. During a second screening, the eligibility of the selected articles was reconsidered by two reviewers (EG and RW) after reading the full text. Any discrepancies and doubts were discussed with all coauthors.

The two co-authors with a technical background (KD and ORR) extracted technical details reported in the articles following the initial list of reporting items (see section 3.1), i.e. items falling into the dimensions of general information and technical data. Among them are aspects related to the personality of the chatbot, embodiment, application technology, intelligence framework, sentiment or emotion recognition (source and algorithm), input and output mode, privacy, etc. Since the chatbots are supposed to interact with persons suffering from depression, we were collecting information on integrated suicide risk assessment methods.

The two co-authors with a background in psychology (EG and RW) extracted the clinical data from the selected articles, i.e. data on the reported study and the depression intervention. Items included characteristics of study participants (number, gender, and age), diagnosis, duration of the intervention. They coded the intervention components incorporated by the chatbots according to the Behavior Change Wheel (BCW) framework (14, 16). The BCW framework was selected because many chatbot-based interventions for depression aim to promote behavior change. The BCW framework helps bridging the gap between technical design and psychological theory by providing a structured framework for categorizing intervention functions for behavior change into nine components: Education (involving techniques to increase knowledge), Persuasion (utilizing communication to prompt action), Incentivization (integrating expectations of reward), Coercion (integrating expectations of punishment), Training (imparting skills), Restriction (employing techniques to limit the opportunity for engaging in the target behavior), Environmental restructuring (involving changes in the physical environment), Modeling (providing examples for people to imitate), and Enablement (increasing means or reducing barriers to enhance capability).

All included studies were included in a qualitative synthesis.

## 3 Results

### 3.1 Initial draft of recommendation list for reporting

The items of our initial draft of the recommendation list were structured along four main dimensions (**Supplementary Material 4**): General information; Chatbot-based depression intervention functions; Technical data; and Study. Items on general information include country, name of the chatbot and version of the chatbot.

As items on the chatbot-based depression intervention functions, we suggest reporting on the 9 intervention components for behavior change that are implemented in the chatbot, as classified in the behavior change wheel (14): education, persuasion, incentivization, coercion, training, restriction, environmental restructuring, modeling, and enabling. In case a technique is implemented in a chatbot, details on how it is realized should be provided. Additional treatment-specific aspects are rather technical and are therefore listed in this category (**Supplementary Material 4**).

As items related to the technical data, we are considering all 18 dimensions proposed by Denecke and May (12) as relevant. These dimensions were extended by 11 additional technical dimensions to address specific features of chatbots for depression treatment (see **Table 1**). This includes the concrete implementation of the natural language processing (NLP), features for personalization and promotion of adherence. Given the fact that some guidelines for developing chatbots and chatbots in healthcare exist already [e.g. DISCOVER (17)], their consideration should be reported in case some have been applied. In terms of the realization of the intervention, it is important to know who initiates the conversation and why. In the context of depression several questionnaires and assessment tools exist. Their integration into the chatbot should be reported. Since depression goes along with a risk of suicidal behavior, it is of relevance to report whether the chatbot integrates a suicide risk assessment and implementation details if integrated. Since chatbots for depression aim at improving symptoms or encourage behavior change, it should be reported how the chatbot is measuring whether symptoms are improving.

**Table 1:** Technical data to be reported.

Agent appearance	Setting	Interaction	Data processing	Use case specific technical aspects
**Personality of CA** 1) simple, 2) complex	**Context** 1) general purpose, 2) domain specific	**Input mode/output mode** 1) written, 2) spoken, 3) visual, 4) hybrid, 4) haptic	**Internet access** 1) online, 2) offline	**How is NLP realized?**
**Embodiment** 1) no, 2) avatar, 3) physical	**Service duration** 1) *ad-hoc* supporters, 2) persistent companions, 3) temporary advisors	**Service channel** 1) smartphone embedded software, 2) social media, 3) website (web-based), 5) smart speaker	**Hosting** 1) local, 2) outsourced, 3) both	**Content creation (Expert-based, other knowledge sources)**
**Application technology** 1) virtual reality, 2) augmented reality, 3) vocal, 4) normal	**Human involvement** 1) diad, 2) triad, 3) quadriad	**Device** 1) PC, 2) mobile device, 3) both, 4) other	**Data exchange with 3^rd^ party device or service** 1) access, 2) storing, 3) both, 4) none	**Suicide risk assessment integrated?**
**Intelligence framework** 1) rule-based, 2) self-learning	**Target user group**	**Language** 1) single language, 2) multi language	**Data privacy** 1) privacy policy, 2) data encryption, 3) both, 4) nothing	**What happens when suicide risk is detected?**
**Sentiment/emotion detection** 1) yes, 2) no	**Initiating conversation** (chatbot, user, user or chatbot)	**Integration mode** 1) stand-alone, 2) part of a system		**Theoretical background**
**Personalization of appearance of chatbot possible**?	**Reasons for initiating conversation**			**How is measured whether symptoms are improving?**
				**Guidelines used for development**
				**Integrated assessment tools and questionnaires**

Technical characteristics fall into 29 dimensions (in bold) grouped along 5 perspectives (first row highlighted in grey).

Another set of reporting items concerns the study, i.e. the technical and clinical evaluation of the chatbot and intervention (see **Table 1**). As study details we suggest reporting at least: Gender, age group, sample size, diagnosis, duration of intervention, desired frequency of chatbot use, endpoints. Regarding technical evaluation, details on the technical evaluation as well as information on user testing should be reported.

An overview of the initial draft of recommendations for improving reporting standards on chatbots for depression is available in the **Supplementary Material 4**.

### 3.2 Findings from the literature review

We identified a total of 215 references in the database search. After removing 15 duplicates, the titles and abstracts of 200 references were screened to determine their eligibility. During this first screening, 158 references were rejected. Subsequently, 41 articles underwent a thorough full-text review. During the full-text review, 18 additional articles were rejected. The list of these 18 rejected articles (18–35), along with the reasons for their rejection, is provided as a **Supplementary Material 3**. The final number of articles included in this review is 23. The flowchart of the selection process is summarized in the **Supplementary Material 2**.

We included a total of 23 articles. These articles were published between 2017 and 2023. 8 papers are authored from researchers from the United States (36–43), 1 paper each from the United Kingdom (44), Italy (45), South Korea (46), Spain (47), Australia (48) and India (49). Two papers each originated from China (50, 51) and Switzerland (52, 53). One paper was authored by a group of researchers from India and United States (54).

A total of 2770 study participants were included in these articles. The 16 articles in which the gender of the study participants is clearly specified have reported the participation of 919 females, 407 males, 1 transgender, 3 non-binary, 2 other, 1 not specified, and 1 genderqueer/androgynous. The mean ages of the study participants ranged between 14.7 for the youngest and 53.2 for the oldest samples.

The studies assessed the presence of depression or depressive symptoms based on users self-reporting or answers to self-administered questionnaires, such as Patient Health Questionnaire (PHQ) in its versions PHQ-9, PHQ-2, PHQ-4, Center for Epidemiologic Studies Depression Scale (CES-D), Edinburgh Postnatal Depression Scale (EPDS), or Patient-Reported Outcomes Measurement Information System (PROMIS). The articles in which the intervention duration was reported tested the chatbots over periods lasting from 2 weeks to 16 weeks.

### 3.3 Technical characteristics of the chatbots

The papers refer to the following chatbots: Wysa (41, 44, 54), mPHA (40, 45), Saathi (49), Sermo (52), Mylo (48), Tess (36, 38, 55), Elena+ (53), Woebot (37, 56, 57), VickyBot (47), Luca (58), XiaoNan (51), XiaoE (50), Smartspeakers (43), Yeonhebot (46), and Pocket Skills (42). All papers which used Wysa are considered to have used the same system, as well as the two papers that used mPHA. Papers which used Woebot or Tess are considered individually - for each paper, the chatbot was adapted in a way that the resulting chatbots have to be considered different systems. They simply rely upon the same framework. Thus, we consider 19 different chatbots. Their technical characteristics are listed in **Supplementary Material 6** and will be described in the following.

Most of the chatbots are domain-specific (18/19), only one was not domain-specific [Smartspeakers (43)]. In 16 systems the interaction takes place between one human and the chatbot (diad, 16/19), while for 3 chatbots two humans are involved in the interaction with the chatbot m-PHA (40, 45), Tess (36, 38, 55), Wysa (41, 44, 54, 59). Duration of the service offered by the chatbot is medium-term (temporary advisor, 8/19), long-term [persistent companion, 4/19, Yeonhebot (46), Pocket Skills (42), XiaoE (50), Woebot (57)] and rarely short-term [*ad hoc* support, 2/19, Smartspeakers (43), XiaoNan (51)]. Service duration is unknown for 5 systems [VickyBot (47), Luca (58), Woebot (37), Saathi (49), Tess (55)].

**Figure 2:**
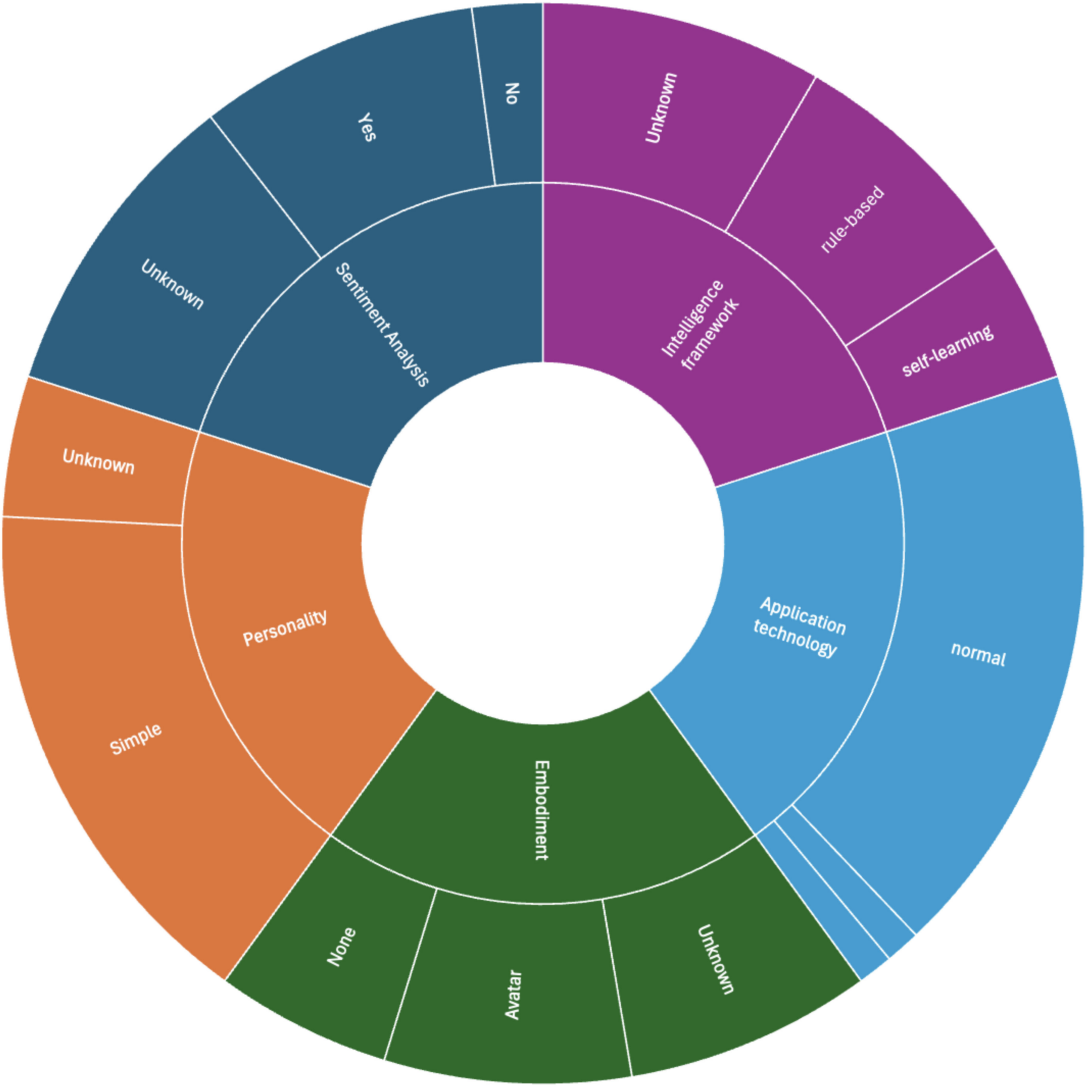
Agent appearance characteristics of the included chatbots (n=19).

**Figure 2** summarizes the agent appearance characteristics. Sentiment analysis is used by 8 chatbots (m-PHA (40, 45), Wysa (41, 44, 54, 59), Sermo (52), Mylo (48), Tess (55), Woebot (37), Tess (38); two chatbots do not use sentiment analysis [Elena+ (53), Luca (58)]; 9 chatbots do not report information on this (Saathi (49), Tess (36), Woebot (56), Smartspeakers (43), VickyBot (47), Yeonhebot (46), Pocket Skills (42), XiaoE (50), Woebot (57). From the 8 chatbots that use sentiment analysis, two are based on pattern matching using lists of terms (48) or emotional dictionaries of SentiWS (52). One Woebot implementation asks explicitly questions on emotions (37) and handles them using decision trees and NLP, but the paper does not specify details on this analysis. XiaoNan used intention classification and emotion recognition models that label the input text with pre-defined intention and emotion tags (51). Again, details are missing.

**Figure 3:**
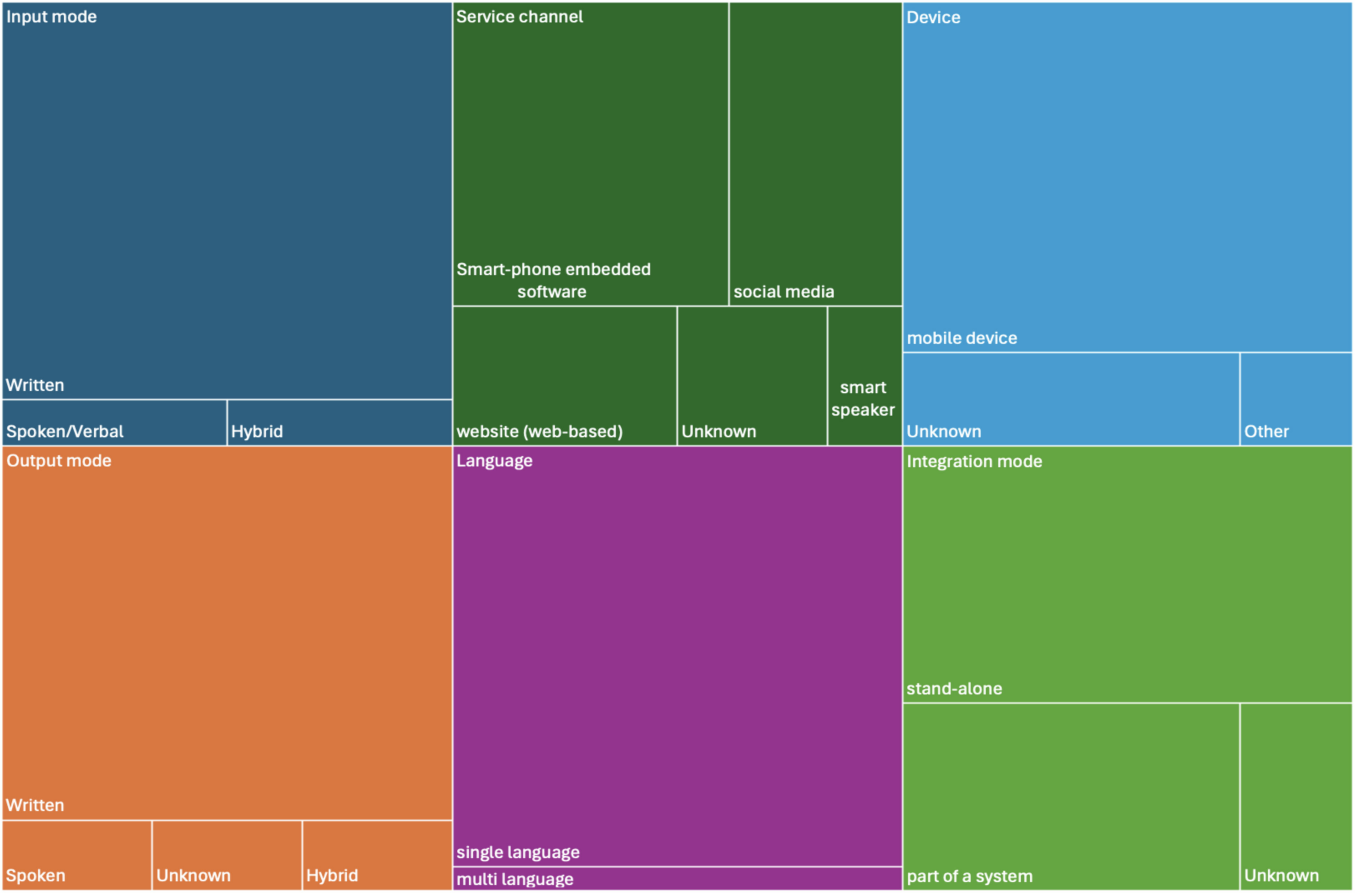
Interaction characteristics of the included chatbots (n=19).

The underlying intelligence framework is rule-based [7/19, Sermo (52), Tess (36), Tess (55), Woebot (56), Woebot (37), Elena+ (53), XiaoNan (51)] or self-learning (4/19, Smartspeakers (43), Mylo (48), m-PHA (40, 45), Wysa (41, 44, 54, 59). With self-learning we refer to chatbot systems that apply machine learning, learn from user interactions for improving performance over time. For 8 chatbots this information could not be extracted. The application technology is normal (17/19) or vocal (1/19, Smartspeakers (43)); for 1 chatbot we could not judge (58). The chatbots have no embodiment [5/19, Sermo (52), Mylo (48), Smartspeakers (43), XiaoNan (51), XiaoE (50)] or use an avatar [7/19, m-PHA (40, 45), Wysa (41, 44, 54, 59), Elena+ (53), Woebot (56), Woebot (37), Yeonhebot (46), Pocket Skills (42)]; embodiment is unknown for the other 7 chatbots. The personality of 15 chatbots is simple (15/19); for 4 chatbots not sufficient information is provided to judge [XiaoNan (51), VickyBot (47), Luca (58), Tess (38)]. Input and output mode is written by the majority of chatbots (17/19); 1 uses spoken/vocal in- and output (43), one is hybrid [written and visual (50)]. 8 chatbots are smartphone-embedded (m-PHA (40, 45), Wysa (41, 44, 54, 59), Sermo (52), Tess (36), Elena+ (53), Woebot (56), VickyBot (47), Woebot (57); 5 are running as part of social media (37, 38, 46, 50, 51); 3 are web-based (42, 48, 58), for 2 it is unknown (49, 55) and one is accessible via smart speakers (43). 15 chatbots are running on mobile devices; for 3 the device is unknown (36, 55, 58) and one uses another device (43). 11 chatbots are stand-alone [mPHA (40, 45), Wysa (41, 44, 54, 59), Saarthi (49), Elena+ (53), Woebot (56), Smartspeakers (43), VickyBot (47), Luca (58), Tess (38), Woebot (57)], 6 are part of a system (Sermo (52), Mylo (48), Woebot (37), XiaoNan (51), Yeonhebot (46), PocketSkills (42), XiaoE (50) and for 2 the integration mode was not reported (36, 55). Except for one chatbot [Elena+ (53)], all others are provided in one language only. The majority (n=13) is provided in English; 1 each in German (52) and Korean (46), 2 in Chinese (50, 51). The multilingual chatbot is available in English and Spanish (53). For one system, no information could be found on the language (47). Interaction characteristics and their distribution among the reviewed chatbots are visualized in **Figure 3**.

Internet access is needed by 7 systems [Smartspeakers (43), Tess (38), XiaoNan (51), XiaoE (50), Luca (58), Yeonhebot (46), Pocket Skills (42)], 1 does not need internet access [Sermo (52)] and for 11 this information is not reported. Hosting information is not provided for 12 chatbots; 2 systems are hosted locally (Wysa (41, 44, 54, 59), Sermo (52) and 5 chatbots are outsourced (Smartspeakers (43), Tess (38), XiaoE (50), Luca (58), Pocket Skills (42)). Three papers report about availability of a data privacy policy (Smartspeakers (43), Wysa (41, 44, 54, 59), Tess (38), Woebot (56)); for 16 chatbots this information is not explicitly reported.

Data exchange is explicitly neglected by 2 chatbots [Wysa (41, 44, 54, 59), Sermo (52)]. 4 systems are storing data [Tess (38), Luca (58), XiaoE (50), Pocket Skills (42)] and 13 systems do not report enough information to judge this [Saarthi (49), mPHA (40, 45), Mylo (48), Tess (36), Tess (55), Elena+ (53), Woebot (56), Smartspeakers (43), Woebot (37), VickyBot (47), XiaoNan (51), Yeonhebot (46), Woebot (57)].

The conversation is initiated by the chatbot (3/19) based on reminders or scheduled times, the user [8/19, Wysa (41, 44, 54, 59), Sermo (52), Mylo (48), Elena+ (53), Smartspeakers (43), Woebot (37), XiaoNan (51), Woebot (57), Luca (58)] or either chatbot or user [2/19, mPHA (40, 45), XiaoE (50)]. For 6 chatbots it remains unclear who initiates the conversation. Content was created by experts [14/19, mPHA (40, 45), Sermo (52), Tess (36), Wysa (41, 44, 54, 59), Tess (55), Elena+ (53), Woebot (56), Woebot (37), VickyBot (47), Tess (38), XiaoNan (51), Yeonhebot (46), Pocket Skills (42), XiaoE (50)], or using other knowledge sources [1/19, Mylo (48)]. It remains unknown for 4 chatbots (Woebot (57), Luca (58), Smartspeakers (43), Saarthi (49).

For five chatbots, some information on the underlying NLP processes and implementations are provided (48, 49, 51, 52, 58). The chatbot Saarthi uses tokenization, stop word removal and feature extraction (ngram, tfxidf) using the Natural language processing toolkit (NLTK) (49). The chatbot SERMO is implemented using simple pattern matching (52). Mylo uses pattern recognition (48); specifically, the system searches key terms like “anxious” and selects corresponding questions. The user is asked for feedback on the reply of the chatbot. In this way, the system learns. Three chatbots are based on the Rasa platform [XiaoE (50), Luca (58), XiaoNan (51)]. XiaoNan’s language understanding module includes three machine learning models for: natural language processing, intention classification, and emotion recognition. The natural language processing model is responsible for collecting necessary information from the users to support the conversation by handling “entities”, “slots”, and “forms”.

Only for 2 chatbots a technical evaluation was reported [Voice assistants (43), Vickybot (47)]. All 4 voice assistants were asked 14 frequently asked questions about postpartum depression, each response was recorded and judged: accurate recognition (assistant correctly transcribes the spoken query), presence of verbal response, clinically appropriate advice provided (43). The clinical appropriateness was below 30% for all 4 devices. For Vickybot, a technical test was performed to evaluate the stability and reliability of the data transmission between devices and servers (47). The tolerance of calls per minute to the server was verified, and logs of bugs were also collected.

Three papers reported about guidelines or recommendations that were used for the chatbot development. They include the health action process approach (HAPA) (53), the American Psychological Association and American Marketing Association safety recommendations (56) as well as design suggestions (46) of Bakker et al. (60), Jain et al. (61) and Grudin and Jacques were taken into consideration for increasing conversations with chatbots (62).

Only three chatbots offer personalization options for the appearance of the chatbot: Users of Mylo can change the colors of their avatar, customize their profile by changing their profile name [Mylo (48)]. Users of Elena+ can change the gender of the bot (choice between Elena and Elliot) (53). Users of Tess can opt for preselected responses similar to existing chatbots (38). This enhances Tess’ capacity to deliver more personalized and integrative interventions. Pocket Skills users can choose an avatar (42).

Eight papers report features for adherence included in the chatbots. Wysa provides daily check-ins, weekly reports, unlocks a premium reward tool, shows a progress roadmap to the user (41). Tess sends reminders, provides emotional support responses and combines words and emojis to be user-friendly (55). Emojis are also used by another implementation of Tess to increase user engagement (38). Elana+ is designed in an interpersonal style (friendly, non-forceful) (53). It includes gamification, i.e. users can receive badges, usage experience expectations are framed. Results can be promoted in social media. An assessment quiz is used to make topic recommendations. Personalized goals related to behavior change can be set. Woebot sends daily push notifications to increase adherence (56). Another version of Woebot tailors content depending on mood state; personalized messages are sent every day (37). Additionally, emojis and animated gifs are used in the chat. Yeonhebot (46) and Pocket Skills (42) integrate gamification elements.

Two chatbots have an integrated suicide risk assessment. VickyBot implements an emergency alert for suicidal thoughts (47); for users who scored item 9 of the PHQ-9 (suicidal thoughts) or if the chatbot detected suicidal inputs using NLP, an alert is sent to the research team and the user was recommended to immediately visit the emergency department and provided with emergency resources (telephone number for health emergencies and nearby hospital locations).

If users reported suicidal or homicidal ideation or indicated a crisis, Tess provided numbers to the national suicide prevention hotline, crisis text line, and 911 (emergency call in the U.S.) and encouraged the user to end the chat and reach out for professional help (38).

### 3.4 Behavior intervention functions incorporated in the chatbots

Training was the most commonly reported function in 19 of the 24 included studies (36–38, 40–42, 44, 45, 47, 49–52, 54–58) with the training intervention commonly incorporated through the use of cognitive behavioral therapy. The next most commonly incorporated intervention function was enablement, which was reported to be incorporated in 15 articles (36, 38, 41, 42, 44, 46–48, 50, 52–55, 57, 59). Examples of enablement functions incorporated to increase capabilities include the use of mindfulness to reduce anxiety or the delivery of specific coping strategies. Education, was also reported in 15 articles (36–38, 42, 44, 47, 48, 50–57), commonly through the delivery of psychoeducation. Persuasion was reported by 14 articles (36–38, 40, 41, 44, 45, 49, 53, 55–57, 59, 63). Examples of how persuasion was incorporated in these chatbots are the use of motivational interviews or by setting goals. Nine articles indicated that the chatbots incorporated functions aimed at promoting environmental restructuring (37, 38, 42, 44, 46, 47, 56, 57, 63), commonly incorporated through the use of push notifications or prompts, which encouraged users to interact with the chatbot; while two studies reported the use of incentivization (Leo et al., 2022b; Ollier et al., 2023), offering users premium rewards or badges after completing activities.

None of the included articles reported using coercion, restrictions, or modeling in their chatbot interventions. **Table 2** summarizes the identified BCW intervention functions reported in the included studies.

**Table 2:** BCW intervention functions adopted in the included studies.

Reference	Chatbot	Training (impart skills)	Enablement (Increase capability)	Education (increase knowledge)	Persuasion (stimulate action)	Environmental restructuring (change in physical environment)	Incentivization (expectations of reward)	Coercion (expectations of punishment)	Restrictions (reduce opportunity to engage in behavior)	Modeling (examples to imitate)
Inkster et al., 2023 (44)	Wysa	X	X	X	X	X				
Beatty et al., 2022 (63)	Wysa	X	X	X	X	X				
Leo et al., 2022b (41)	Wysa	X	X		X		X			
Inkster et al., 2018 (59)	Wysa	X	X		X					
Suharwardy et al., 2023 (57)	Woebot	X	X	X	X	X				
Nicol et al., 2022 (56)	Woebot	X		X	X	X				
Fitzpatrick et al., 2017 (37)	Woebot	X		X	X	X				
Klos et al., 2021 (55)	Tess	X	X	X	X					
Dosovitsky et al., 2020 (36)	Tess	X	X	X	X					
Fulmer et al., 2018 (38)	Tess	X	X	X	X	X				
Leo et al., 2022 (40)	mPHA	X			X					
Danieli et al., 2021 (45)	mPHA	X			X					
Anmelia et al., 2023 (47)	VickyBot	X	X	X		X				
Ollier et al., 2023 (53)	Elena+		X	X	X		X			
Rani et al., 2023 (49)	Saathi	X			X					
Wrightson-Hester et al., 2023 (48)	Mylo		X	X						
He et al., 2022 (50)	XiaoE	X	X	X						
Liu et al., 2022 (51)	XiaoNan	X		X						
Burger et al., 2022 (58)	Luca	X								
Yang et al., 2021 (43)	Smartspeakers									
Ryu et al., 2020 (28)	Yeonhebot		X			X				
Denecke et al., 2020 (52)	Sermo	X	X	X						
Schroeder et al., 2018 (42)	Pocket Skills	X	X	X		X				

## 4 Discussion

### 4.1 Reflections on the application of the reporting items

In this paper, we formulated recommendations for items to be considered when reporting about scientific studies related to depression involving a chatbot. The reporting items fall into four dimensions: General information; chatbot-based depression intervention functions; Technical data, and Study. A checklist is provided as **Supplementary Material 5**. By applying this within a literature review, we had to recognize that a lot of information was missing specifically on the involvement of natural language processing, data privacy handling, data exchange with third party providers, and hosting. This hampers comparison and reproducibility of systems. However, our results correspond to the results described by Denecke et al. who tried to extract information on 173 chatbots using the technical-oriented taxonomy (64) that is part of our reporting items. Even for the chatbot that was approved by the U.S. Food and Drug Administration (WoeBot (37, 56, 57), no information on the use of NLP is described in the published article. It might be that researchers believe that the information is available somewhere else. However, for the sake of transparency, we would expect a description of basic technical implementation details on a chatbot in each publication.

Additionally, details on a technical evaluation are not reported in many papers. This raises the question whether chatbots for depression treatment are sufficiently evaluated from a technical perspective before being used in clinical trials. Technically weak chatbots might risk patient safety, even in clinical trials, and interacting with a technically weak system might also impact future acceptance of such systems. With the increased use of generative artificial intelligence, a lack of a comprehensive technical evaluation of a chatbot could lead to serious risks for patient safety (65). The question arises how it can be ensured that provided information is correct and misinformation or mistreatment is avoided. Meyrowitsch et al. suggest that companies providing chatbots based on artificial intelligence serve as gatekeepers and ensure that the content provided is correct, but that also patients have to be enabled to judge the quality of an information source (66). For evaluating health chatbots, there are validated frameworks available such as the one presented by Denecke et al. (12, 67). Although not specifically developed for chatbot-based interventions for depression, such framework guides to relevant aspects to be considered when evaluating these systems.

In this paper, we focused on studies related to chatbots for depression. Depression can in some cases lead to suicide (3). However, only 2 systems involve a suicide risk assessment and reactions on detected suicide-related events. The FDA approved system is not among them, probably the scope of the system was set in a way that it is not necessary. Again, lack of risk detection integrated in such tools might raise significant safety concerns for users. However, we cannot be sure whether the papers included in the review simply do not report about integrated suicide risk detection or whether they really do not integrate it. It would be useful not only from a research perspective, but also from a practical perspective to know, whether a system integrates suicide risk assessment or not as this information impacts on the relevance of accompanying patient safety measurements. From a practical perspective, this information could impact on the recommendation behavior of health professionals, as they could better judge the patient safety risks when using the application with having such information or even consider a closer supervision when no such assessment is integrated. Potential risks such as risk of dependency, potential for misinformation, or delay in help seeking should be reflected already during the development of digital health interventions as was suggested in recent research (68, 69). This would contribute to patient safety - only risks or adverse events that are considered can be recognized and handled appropriately. Additionally, consideration of ethical frameworks such as the principles of biomedical ethics of Beauchamp and Childress (70) or the guidelines of the American Psychological Association (APA (71)) could be reported. The framework by Beauchamp and Childress would provide a foundational guide for ensuring that chatbot interventions promote patient well-being, avoid harm, respect patient autonomy, and provide fair access to care. APA guidelines emphasize the importance of informed consent, confidentiality, and the responsible use of technology in mental health interventions.

We believe that reporting technical details is important even in papers on clinical trials that exploit a chatbot. From our review and specifically from the missing information, the assumption arises for example that the full potential of NLP is not yet used in the implementations of chatbots for depression. NLP techniques can be used to analyze patterns in user language and behavior over time. By tracking changes in language usage or sentiment, the chatbot can identify potential barriers to behavior change and offer targeted interventions to address them. While depression involves depressed sentiments and emotions, sentiment and emotion analysis is not yet used by all chatbots. Sentiment analysis can be used to analyze the sentiment and emotions expressed in user messages, allowing the chatbot to gauge the user’s emotional state (72). Based on the sentiment analysis, the chatbot can tailor responses to provide appropriate emotional support or encouragement.

From the papers, we did not learn much about whether and how contextual understanding is realized. Advanced NLP models can enable chatbots to understand the context of conversations and adapt their responses accordingly. By taking into account the context of previous interactions, the chatbot can provide more relevant and effective support for behavior change (73).

The lack of technical details of the chatbots also hamper to aggregate best practices in the development of digital health interventions. From our review, we cannot draw any conclusions on which NLP techniques can be successfully used to implement specific behavior change techniques. From a development perspective, this information would be useful to be able to select technologies for developing efficient and effective digital health solutions.

Information on the underlying psychological models were more complete. However, while all papers mentioned the theories, they based their approach on, not all described in detail how these theories were applied in practice. Psychological interventions generally prioritize alleviating distress and enhancing mental well-being, rather than exacerbating the negative emotions already characteristic of depression. The inclusion of coercion as an intervention function might heighten feelings of failure or shame in individuals with depression, given their higher sensitivity to punishment (74). Appropriately, none of the chatbots incorporated coercion into their interventions. It is not obvious, following our present review, whether the clinical data that exists at this stage is sufficient for making recommendations regarding the use of chatbots for patients with clinical depression. A main challenge with the studies we have identified and included is that the chatbots rarely have been tested on clinical populations, i.e. samples of people that have been evaluated according to a diagnostic standard (such as the ICD-10 or DSM-V). Moreover, the control conditions used in many of the included studies, such as ‘bibliography’ are not clinically relevant. It would not be an alternative for a clinically depressed patient to only read about depression (‘bibliotherapy’). Viable and clinically relevant alternatives would be psychotherapy, such as therapy, and/or medication, such as selective serotonin reuptake inhibitors (SSRIs). This means that chatbot treatment for depression should be compared to established and evidence-based treatments - if chatbot treatment is meant to be an alternative to these established treatments. It is understandable that most studies carried out at this early stage do not meet criteria for being used as a basis for clinical guidelines. The studies required many resources in terms of clinically skilled people, time and money. Investing such resources in the testing of a chatbot would probably depend on the expectation that the chatbot could be monetized on a large scale, which might not be the case at this stage of chatbot development.

### 4.2 Strengths and limitations

This study provides the first draft of recommended reporting items for studies on chatbots for depression. The initial list of items was collected based on the authors’ experiences and existing research. We might have missed relevant aspects. In future work, we will conduct a Delphi study, to find consensus on the reporting items. However, we believe that the recommendations in their current form may be already useful and their application by researchers could result in practical feedback that would enrich also Delphi panel results.

Although the review was carefully designed, there is a risk of publication bias since we did not involve a general web search to find chatbots for depression treatment that have not been published. However, we believe that chatbots used for treating a medical condition such as depression need careful assessment of efficacy and risks with results from clinical trials published in scientific papers as this forms the basis for evidence-based medicine. Company websites may describe non-reproducible information, ignoring scientific standards which make them less reliable. We excluded studies that presented chatbots addressing depression together with other mental health conditions (such as anxiety). Therefore, we might have missed some relevant information.

The included studies did not necessarily involved individuals that were clinically diagnosed with depression. This reflects the current state of research where chatbot-based applications are tested with populations that self-diagnosed a specific disease.

We considered the four papers referring to the Wysa chatbot as referring to one system and also the two papers on the m-PHA chatbot when reporting technical details. But we extracted the BCW functions from all papers. It occurred that articles referring to the same chatbot do not always report on exactly the same amount of BCW functions.

## 5 Conclusions

In this paper, we developed a list of items to be reported when describing studies on chatbot-based interventions for depression. From an application of our recommendation list, we can conclude that some existing articles on chatbot-based interventions for depression have better reporting on these functions than others. Reporting technical details is important even in papers on clinical trials that utilize chatbots in order to allow reproducibility and advance this field. Studies on chatbots for depression can improve reporting by specifically adding more technical details and chatbot evaluation. Future work could obtain expert consensus on the recommended reporting items for chatbot-based interventions for depression. To allow for progress in this field, including replications, researchers should clearly report the incorporated functions. There are reporting checklists such as the CONSORT‐EHEALTH (75), that provide general guidance for reporting digital health intervention, but they do not address technical or chatbot-specific dimensions in detail. Integrating our reporting items into such checklists would complement existing frameworks by offering targeted guidance on chatbot-based interventions, thereby supporting researchers and promoting transparent reporting. Our list of recommendations might help identify effective chatbot features for depression interventions; it can improve the integration of chatbots into mental health interventions and facilitate evidence-based advances in the field. Beyond, it supports the systematic evaluation of chatbot effectiveness, safety and patient outcomes, contributing to better informed clinical practice and policymaking in mental health care.

## Data Availability

The original contributions presented in the study are included in the article/**Supplementary Material**. Further inquiries can be directed to the corresponding author.
