# Supplementary material for: Persuasive chatbot-based interventions for depression: a list of recommendations for improving reporting standards
Source: Front Psychiatry. 2025 Jun 19;16:1429304. doi: 10.3389/fpsyt.2025.1429304 (PMC12222314; doi:10.3389/fpsyt.2025.1429304)
Supplement: Supplementary file 1 [file DataSheet1.docx]

Supplementary Material

**Persuasive chatbot-based interventions for depression: A list of recommendations for improving reporting standards**

**Kerstin Denecke, Octavio Rivera Romero, Rolf Wynn^*^, Elia Gabarron**

*** Correspondence:** Rolf Wynn: rolf.wynn@uit.no

# Supplementary Material 1: Full search strategy

Supplementary Table 1: Full search strategy

| Search engine | Search string | Results |
| --- | --- | --- |
| PubMed | ("chatbot"[Title/Abstract] or "conversational agent"[Title/Abstract] or "intelligence agent"[Title/Abstract] or "virtual assistant"[Title/Abstract]) and ("depression"[Title/Abstract] or "Depressive disorder"[Title/Abstract] or "dysthymia"[Title/Abstract] or "affective disorder"[Title/Abstract] or "dysthymic disorder"[Title/Abstract]) | 100 |
| ACM | Title:(("Depression" OR "Depressive disorder" OR "Dysthymia" OR "Affective disorder" OR "Dysthymic disorder") AND ("chatbot" OR "conversational agent" OR "intelligent agent" OR "virtual assistant")) OR Abstract:(("Depression" OR "Depressive disorder" OR "Dysthymia" OR "Affective disorder" OR "Dysthymic disorder") AND ("chatbot" OR "conversational agent" OR "intelligent agent" OR "virtual assistant")) | 9 |
| IEEExplore | (((("Document Title":"Depression" OR "Depressive disorder" OR "Dysthymia" OR "Affective disorder" OR "Dysthymic disorder") AND ("Document Title":"chatbot" OR "conversational agent" OR "intelligent agent" OR "virtual assistant")) OR (("Abstract":"Depression" OR "Depressive disorder" OR "Dysthymia" OR "Affective disorder" OR "Dysthymic disorder") AND ("Abstract":"chatbot" OR "conversational agent" OR "intelligent agent" OR "virtual assistant")))) | 50 |
| PsycInfo | ((title: "Depression" OR title: "Depressive disorder" OR title: "Dysthymia" OR title: "Affective disorder" OR title: "Dysthymic disorder") AND (title: "chatbot" OR title: "conversational agent" OR title: "intelligent agent" OR title: "virtual assistant")) OR ((abstract: "Depression" OR abstract: "Depressive disorder" OR abstract: "Dysthymia" OR abstract: "Affective disorder" OR abstract: "Dysthymic disorder") AND (abstract: "chatbot" OR abstract: "conversational agent" OR abstract: "intelligent agent" OR abstract: "virtual assistant")) | 30 |
| CINAHL | TI (("Depression" OR "Depressive disorder" OR "Dysthymia" OR "Affective disorder" OR "Dysthymic disorder") AND ("chatbot" OR "conversational agent" OR "intelligent agent" OR "virtual assistant")) OR (AB (("Depression" OR "Depressive disorder" OR "Dysthymia" OR "Affective disorder" OR "Dysthymic disorder") AND ("chatbot" OR "conversational agent" OR "intelligent agent" OR "virtual assistant"))) | 26 |

Search date: January 8th 2024

# Supplementary Material 2: PRISMA flowchart of the selection process


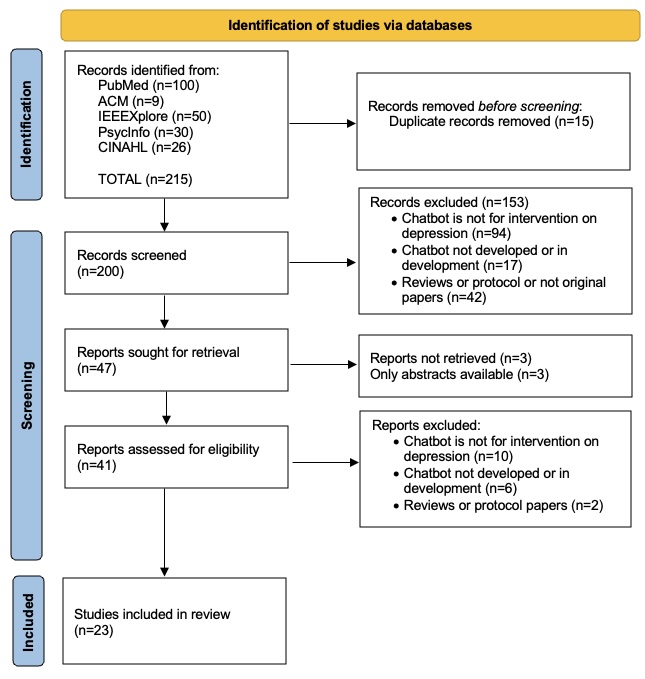


Supplementary Figure 1. PRISMA flowchart of the selection process

# Supplementary Material 3: Excluded articles

Articles excluded during the full-text review and reasons for exclusion

**The chatbot is not used for an intervention for any of the following conditions: Depression, Depressive disorder; Dysthymia; Affective disorder; Dysthymic disorder**

·       Emotion Guru: A Smart Emotion Tracking Application with AI Conversational Agent for Exploring and Preventing Depression (1)

·       Modeling an ambient agent to support depression relapse prevention (2)

·       Providing Self-Led Mental Health Support Through an Artificial Intelligence–Powered Chat Bot (Leora) to Meet the Demand of Mental Health Care (3)

·       Smart Solutions to Keep Your Mental Balance (4)

·       Therapy Chatbot Powered by Artificial Intelligence:  A Cognitive Behavioral Approach (5)

·       Towards Motivational and Empathetic Response Generation in Online Mental Health Support (6)

·       Combating Depression in Students using an Intelligent ChatBot: A Cognitive Behavioral Therapy (7)

·       Experiences of a Speech-enabled Conversational Agent for the Self-report of Well-being among People Living with Affective Disorders_ An In-the-Wild Study (8)

·       Evaluation of chatbot-delivered interventions for self-management of depression: Content analysis (9)

·       Engagement, Satisfaction, and Mental Health Outcomes Across Different Residential Subgroup Users of a Digital Mental Health Relational Agent-Exploratory Single-Arm Study (10)

**The chatbot is not described or it has not been developed**

·       A Personalized Support Agent for Depressed Patients (11)

·       “I don’t know what you mean by ‘I am anxious”’: A New Method for Evaluating Conversational Agent Responses to Standardized Mental Health Inputs for Anxiety (12)

·     A Digital Lifestyle Program for Psychological Distress, Wellbeing and Return-to-Work: A Proof-of-Concept Study (13)

·       Co-developing a mental health and wellbeing chatbot with and for young people (14)

·       Development of a chatbot for depression: adolescent perceptions and recommendations (15)

·       Digital Psychiatry – Curbing Depression using Therapy Chatbot and Depression Analysis (16)

**Reviews, study protocols, comments, patents and white papers**

·       CareBot : A Mental Health ChatBot (17)

·       Optimizing Students’ Mental Health and Academic Performance: AI-Enhanced Life Crafting (18)

References

1. Cuylenburg HC van, Ginige TNDS. Emotion Guru: A Smart Emotion Tracking Application with AI Conversational Agent for Exploring and Preventing Depression. 2021 International Conference on UK-China Emerging Technologies (UCET). 2021;1–6.

2. Aziz AA, Klein MCA, Treur J. Modeling an Ambient Agent to Support Depression Relapse Prevention. 2009 IEEE/WIC/ACM International Joint Conference on Web Intelligence and Intelligent Agent Technology. 2009;3:335–40.

3. van der Schyff E, Ridout B, Amon K, Forsyth R, Campbell A. Providing Self-Led Mental Health Support Through an Artificial Intelligence-Powered Chat Bot (Leora) to Meet the Demand of Mental Health Care. Journal of medical Internet research. 2023 Jun;25:e46448.

4. Gifu D, Pop E. Smart Solutions to Keep Your Mental Balance. Procedia computer science. 2022;214:503–10.

5. Shetty M, Shah P, Shah K, Shinde V, Nehete S. Therapy Chatbot Powered by Artificial Intelligence: A Cognitive Behavioral Approach. 2023 International Conference in Advances in Power, Signal, and Information Technology (APSIT). 2023;457–62.

6. Saha T, Gakhreja V, Das AS, Chakraborty S, Saha S. Towards Motivational and Empathetic Response Generation in Online Mental Health Support - Proceedings of the 45th International ACM SIGIR Conference on Research and Development in Information Retrieval. Proceedings of the 45th International ACM SIGIR Conference on Research and Development in Information Retrieval. 2022;2650–6.

7. Patel F, Thakore R, Nandwani I, Bharti SK. Combating Depression in Students using an Intelligent ChatBot: A Cognitive Behavioral Therapy. 2019 IEEE 16th India Council International Conference (INDICON). 2019;1–4.

8. Maharjan R, Doherty K, Rohani DA, Bækgaard P, Bardram JE. Experiences of a speech-enabled conversational agent for the self-report of well-being among people living with affective disorders: An in-the-wild study. ACM Transactions on Interactive Intelligent Systems. 2022 Jun;12(2):1–29.

9. Martinengo L, Lum E, Car J. Evaluation of chatbot-delivered interventions for self-management of depression: Content analysis. Journal of affective disorders. 2022 Dec;319:598–607.

10. Forman-Hoffman V, Pirner M, Flom M, Kirvin-Quamme A, Durden E, Kissinger J, et al. Engagement, Satisfaction, and Mental Health Outcomes Across Different Residential Subgroup Users of a Digital Mental Health Relational Agent: Exploratory Single-Arm Study. JMIR formative research. 2023 Sep;7:e46473.

11. Kop R, Hoogendoorn M, Klein MCA. A Personalized Support Agent for Depressed Patients: Forecasting Patient Behavior Using a Mood and Coping Model. 2014 IEEE/WIC/ACM International Joint Conferences on Web Intelligence (WI) and Intelligent Agent Technologies (IAT). 2014;3:302–9.

12. Eagle T, Blau C, Bales S, Desai N, Li V, Whittaker S. “I Don’t Know What You Mean by `I Am Anxious’”: A New Method for Evaluating Conversational Agent Responses to Standardized Mental Health Inputs for Anxiety and Depression. ACM Trans Interact Intell Syst [Internet]. 2022 Jul;12(2). Available from: https://doi.org/10.1145/3488057

13. Brinsley J, Singh B, Maher C. A Digital Lifestyle Program for Psychological Distress, Wellbeing and Return-to-Work: A Proof-of-Concept Study. Archives of physical medicine and rehabilitation. 2023 Nov;104(11):1903–12.

14. Grové C. Co-developing a Mental Health and Wellbeing Chatbot With and for Young People. Front Psychiatry. 2020;11:606041.

15. Dosovitsky G, Bunge E. Development of a chatbot for depression: Adolescent perceptions and recommendations. Child and Adolescent Mental Health. 2023 Feb;28(1):124–7.

16. Sharma B, Puri H, Rawat D. Digital Psychiatry - Curbing Depression using Therapy Chatbot and Depression Analysis. 2018 Second International Conference on Inventive Communication and Computational Technologies (ICICCT). 2018;627–31.

17. Crasto R, Dias L, Miranda D, Kayande D. CareBot: A Mental Health ChatBot. 2021 2nd International Conference for Emerging Technology (INCET). 2021;1–5.

18. Dekker I, De Jong EM, Schippers MC, De Bruijn-Smolders M, Alexiou A, Giesbers B. Optimizing Students’ Mental Health and Academic Performance: AI-Enhanced Life Crafting. Front Psychol. 2020;11:1063.

# Supplementary Material 4: Overview of the initial draft of recommendations for improving reporting standards on chatbots for depression

| **General information** | **Chatbot-based depression intervention functions* that are used and how** | **Technical data** | **Study details** |
| --- | --- | --- | --- |
| - - Name and version of the chatbot      - - Country | - - Education (involving techniques to increase knowledge)      - - Persuasion (utilizing communication to prompt action)      - - Incentivization (integrating expectations of reward)      - - Coercion (integrating expectations of punishment)      - - Training (imparting skills)      - - Restriction (employing techniques to limit the opportunity for engaging in the target behavior) - - Environmental restructuring (involving changes in the physical environment)      - - Modeling (providing examples for people to imitate) - - Enablement (increasing means or reducing barriers to enhance capability) | - - Concrete implementation of NLP - - Detailed information on chatbot implementation including data privacy and security aspects and technical quality - - Features for personalization and promotion of adherence - - Information on applied guidelines for developing the chatbot - - Information on who initiates the conversation with the chatbot and why      - - Information on integrated depression questionnaires and clinical evidence - - Information on how the chatbot is measuring change in symptoms - - Information on integrated suicide risk assessment | - - Clinical evaluation of the chatbot and intervention: Study participants (gender, age group, sample size, diagnosis); duration of intervention; desired frequency of chatbot use; endpoints. - - Details on the technical evaluation and user testing |

* By drawing in the Behaviour Change Wheel framework

# Supplementary Material 5: Reporting checklist for chatbot-based depression interventions

# 1. General Information

• Chatbot Name & Version: Clearly state the name and version of the chatbot used.

• Country: Indicate the country where the study or chatbot deployment took place.

# 2. Chatbot-Based Depression Intervention Functions

• Education: Techniques used to increase users’ knowledge about depression.

• Persuasion: Communication strategies used to prompt positive action or behavior change.

• Incentivization: Mechanisms that offer rewards (real or perceived) to encourage engagement.

• Coercion: Strategies that involve perceived punishment or negative consequences.

• Training: How the chatbot teaches specific skills (e.g., CBT, mindfulness).

• Restriction: Methods limiting the opportunity to engage in undesired behavior.

• Environmental Restructuring: Changes in the user’s physical or digital environment facilitated by the chatbot.

• Modeling: Providing behavioral examples for users to imitate.

• Enablement: Efforts to reduce barriers and increase the user’s capacity to improve mental health.

# 3. Technical Data

• NLP Implementation: Description of how Natural Language Processing (NLP) is concretely implemented in the chatbot.

• Data Privacy & Security: Information on how user data is handled, stored, and protected.

• Personalization Features: Description of how the chatbot adapts to individual users and promotes adherence.

• Development Guidelines: Frameworks or clinical guidelines followed in chatbot development.

• Initiation Dynamics: Who starts the conversation (user or chatbot) and under what conditions.

• Depression Measures: Depression questionnaires or diagnostic tools integrated into the chatbot.

• Symptom Change Assessment: How symptom changes are tracked and evaluated (e.g., pre/post survey, real-time analysis).

• Suicide Risk Assessment: Any mechanisms included for detecting and responding to suicide risk.

# 4. Study Details

• Participant Information: Sample size, gender distribution, age group(s), and diagnostic criteria.

• Intervention Duration: Total length of the intervention and desired chatbot usage frequency.

• Study Endpoints: Primary and secondary outcomes used to evaluate effectiveness.

• Technical Evaluation: Tests of chatbot performance, reliability, uptime, etc.

• User Testing: Usability studies, user feedback, satisfaction ratings, etc.

# Supplementary Material 6: Technical characteristics of the reviewed chatbots

| **Chatbot** | **Personali-ty of Chatbot** | **Embo-diment** | **Application technology** | **Intelligence Frame-work** | **Sentiment or emotion recognition** | **Context** | **Service duration** | **Human involvement** | **Input mode** | **Output mode** | **Service channel** | **Device** | **Language** | **Integration mode** | **Internet access** | **Hosting** | **Data exchange with third party devices** | **Data privacy** | **Content created** | **Initiating conversation** |
| --- | --- | --- | --- | --- | --- | --- | --- | --- | --- | --- | --- | --- | --- | --- | --- | --- | --- | --- | --- | --- |
| m-PHA (40,45) | Simple | Avatar | Normal | Self-learning | Yes | Domain-specific | Temporary advisor | Triad | Written | Written | Smartphone-embedded | Mobile device | English | Stand-alone | Unknown | Unknown | Unknown | Unknown | Expert-based | Chatbot, user |
| Wysa (41,44,59,63) | Simple | Avatar | Normal | Self-learning | Yes | Domain-specific | Temporary advisor | Triad | Written | Written | Smartphone-embedded | Mobile device | English | Stand-alone | Unknown | Local | None | Unknown | Expert-based | User |
| Saathi (49) | Simple | Unknown | Normal | Unknown | Unknown | Domain-specific | Unknown | Diad | Written | Written | Unknown | Mobile device | English | Stand-alone | Unknown | Unknown | Unknown | Unknown | Unknown | Unknown |
| Sermo (52) | Simple | Non | Normal | Rule-based | Yes | Domain-specific | Temporary advisor | Diad | Written | Written | Smartphone-embedded | Mobile device | German | Part of a system | Offline | Local | None | Unknown | Expert-based | User |
| Mylo (48) | Simple | None | Normal | Self-learning | Yes | Domain-specific | Temporary advisor | Diad | Written | Written | Web-based | Mobile device | English | Stand-alone | Unknown | Unknown | Unknown | Unknown | Other knowledge source | User |
| Tess (36) | Simple | Unknown | Normal | Rule-based | Unknown | Domain-specific | Temporary advisor | Diad | Written | Written | Smartphone-embedded | Unknown | English | Unnown | Unknown | Unknown | Unknown | Unknown | Expert-based | Unknown |
| Tess (55) | Simple | Unknown | Normal | Rule-based | Yes | Domain-specific | Unknown | Diad | Written | Written | Unknown | Unknown | English | Unknown | Unknown | Unknown | Unknown | Unknown | Expert-based | Unknown |
| Elena+ (53) | Simple | Avatar | Normal | Rule-based | No | Domain-specific | Temporary advisor | Diad | Written | Written | Smartphone-embedded | Mobile device | English, Spanish | Stand-alone | Unknown | Unknown | Unknown | Unknown | Expert-based | User |
| Woebot (56) | Simple | Avatar | Normal | Rule-based | Unknown | Domain-specific | Temporary advisor | Diad | Written | Written | Smartphone-embedded | Mobile device | English | Stand-alone | Unknown | Unknown | Unknown | Privacy policy | Expert-based | Unknown |
| Smartspeakers (43) | Simple | None | Vocal | Self-learning | Unknown | Unknown | Ad hoc supporter | Diad | Spoken | Spoken | Smart speaker | Other | English | Stand-alone | Online | Outsourced | Unknown | Privacy policy | Unknown | User |
| Woebot (37) | Simple | Avatar | Normal | Rule-based | Yes | Domain-specific | Unknown | Diad | Written | Written | Social media | Mobile device | English | Part of a system | Unknown | Unknown | Unknown | Unknown | Expert-based | User |
| VickyBot (47) | Unknown | Unknown | Normal | Unknown | Unknown | Domain-specific | Unknown | Diad | Written | Unknown | Smartphone-embedded | Mobile device | Unknown | Stand-alone | Unknown | Unknown | Unknown | Unknown | Expert-based | Unknown |
| Tess (38) | Unknown | Unknown | Normal | Unknown | Yes | Domain-specific | Temporary advisor | Triad | Written | Written | Social media | Mobile device | English | Stand-alone | Online | Outsourced | Storing | Privacy policy | Expert-based | Chatbot |
| XiaoNan (51) | Unknown | None | Normal | Rule-based | Yes | Domain-specific | Ad hoc supporter | Diad | Written, Spoken | Written | Social media | Mobile device | Chinese | Part of a system | Online | Unknown | Unknown | Unknown | Expert-based | User |
| Luca (58) | Unknown | Unknown | Unknown | Unknown | No | Domain-specific | Unknown | Diad | Written | Written | Web-based | Unknown | English | Stand-alone | Online | Outsourced | Storing | Unknown | Unknown | User |
| Yeonhebot (46) | Simple | Avatar | Normal | Unknown | Unknown | Domain-specific | Persistant companion | Diad | Written | Written | Social media | Mobile device | Korean | Part of a system | Online | Unknown | Unknown | Unknown | Expert-based | Chatbot |
| Pocket Skills (42) | Simple | Avatar | Normal | Unknown | Unknown | Domain-specific | Persistant companion | Diad | Written | Written | Web-based | Mobile device | English | Part of a system | Online | Outsourced | Storing | Unknown | Expert-based | Chatbot |
| XiaoE (50) | Simple | None | Normal | Unknown | Unknown | Domain-specific | Persistant companion | Diad | Written | Written, Visual | Social media | Mobile device | Chinese | Part of a system | Online | Outsourced | Storing | Unknown | Expert-based | Chatbot, user |
| Woebot (57) | Simple | Unknown | Normal | Unknown | Unknown | Domain-specific | Persistant companion | Diad | Written | Written | Smartphone-embedded | Mobile device | English | Stand-alone | Unknown | Unknown | Unknown | Unknown | Unknown | User |
